# Supplementary material for: Magnitude, resistance profiles, and risk factors of intestinal parasites and enteric bacteria among food handlers in West Guji Zone, Ethiopia
Source: PLoS One. 2026 Feb 27;21(2):e0342489. doi: 10.1371/journal.pone.0342489 (PMC12948045; doi:10.1371/journal.pone.0342489)
Supplement: Attachment — Submitted filename: Rebuttal letter.pdf [file pone.0342489.s003.pdf]

Response letter

We are pleased to submit the revised version of our manuscript, together with the responses to the comments. We would like to thanks for the constructive comments, which truly improved the level of the manuscript. We believe that we addressed all the comments/suggestion. After careful consideration of the comments, we have outlined point by point response together with the comments (copy and pasted), which is also uploaded as separated file. The specific changes in the main text are highlighted in yellow color. We hope the revised manuscript will received your favorable consideration for publication

Response for Reviewer 2

1) The title suggests a broad focus ("Microbial threats"), but the study only analyzes two bacteria (Salmonella and Shigella) and intestinal parasites.It is recommended to adjust the title or justify why only these agents were considered.

   Response: - Than you for your comment we have adjust title to "Magnitude, Resistance Profiles, and Risk Factors of Intestinal Parasites and Enteric Bacteria among Food Handlers in West Guji Zone, Ethiopia"

2) The introduction needs more microbiological argumentation on: Why Salmonella and Shigella are priorities in the area; The global and local importance of antimicrobial resistance; Previous models or evidence of risk in food handlers in Ethiopia.
   Response: - Thank you for your valuable comment. I have incorporated the suggested information into the main manuscript.

3) There are onconsistencies detected:

-Inconsistency of percentages and counts in several parts (e.g., the abstract mentions 39.2% and then 52.5% as the total prevalence).

Response: - Thank you for your comment what we wanted to say was the prevalence of both bacterial and parasite (52.5%). And 39.2% is the prevalence of parasite alone. However based on your comment as well as other reviewer comment we have removed sentence which is prevalence of both bacterial and parasite (52.5%).

- The wording in Results sometimes mixes descriptive observations with interpretive analysis (which should go in Discussion).

Response: - Thank you for your best comment I have removed them from result section

- In some paragraphs, the term “health status” is used interchangeably to refer to Parasitic infection, Bacterial infection and Hygiene practices; This is conceptually incorrect and confusing.

Response:-Thank you for your excellent insight. In this study, we assessed the health status of food handlers by examining both bacterial and parasitic infections, as well as hygienic practices to identify potential risk factors. I have reviewed my document and could not find any instance where these variables were used interchangeably. They were analyzed independently, and they are independent and we can’t use interchangeably as you commented. Please can you located where it was happened?

4) Key references on microbiology and antimicrobial resistance of Salmonella/Shigella are missing; please revise WHO Global Antimicrobial Resistance Surveillance System (GLASS), Current CLSI M100 (2023–2025), Regional epidemiological data from FAO/WHO, Some articles cited are duplicates or irrelevant to microbiology, Several references are incorrectly cited (format, incomplete information).

Response:-Thank you I have suggestion I have incorporate it

5) There are also some methodological issues:

- Lack of clarity regarding what method was used to identify E. histolytica/dispar? (E. histolytica cannot be identified by microscopy alone); "Bacteria” seems to refer exclusively to Salmonella and Shigella, but the text uses “bacteria” as if it were general, this should be explicitly clarified.

Response: - Thank you for your critical comment for parasite identification. Thus in our case it should be E. histolytica/dispar complex. We accepted and updated through the document. Besides, we have edited issue of using general term “bacteria” with *Prevalence of salmonellosis and shigellosis*

- Samples for bacteria, transport in Cary Blair at 4°C is correct, but the maximum time allowed according to CLSI should be indicated. It is not specified whether late samples were discarded.

Response: - This is very great concern**.** The sample was transported within one hour of collection and analyzed within 48 hours.

- Antimicrobials tested, the panel is limited and does not include antibiotics recommended by CLSI for Salmonella/Shigella (Azithromycin, Cefotaxime/cefixime, Carbapenems (in surveillance studies), Doxycycline is not a drug recommended for Shigella by CLSI.

Response: - This is very great concern. The comments is accepted

- Lack of molecular methodology, it is desirable molecular confirmation (PCR) of serotypes in Salmonella and testing for common resistance genes (such as blaTEM, gyrA, etc.).

Response: - This very great suggestion**.** We couldn't afford and get access to PCR for our study and it may be included under limitation of the study.

6) The results are useful, but not sufficient for broad conclusions such as "high prevalence" or "public policy recommendations." The combined prevalence (52.5%) mixes parasites + bacteria, which is not a valid epidemiological metric. Also, there ar not presented distribution by age/sex for each agent. Could be useful bar charts or trend graphs. You can also include a heatmap for Parasite load (intensity), only presence/absence. Regarding the antimicrobials there is a lack of presentation of mean halos or standard deviation and no confirmation of whether current CLSI cut-off points were applied.

Response: - Thank you for your comment. Conclusion was edited. I have removed combining bacteria and parasite prevalence. However, we haven't presented distribution by age/sex for each agent group but we have make sex/age independent variable which may affect the health outcome of food handles. For AMS test the CLSI cut-off points were applied.

7) The discussion section shows important issues:

- Repeats many results instead of interpreting them.

Response: - some key finding was written on discussion for reader to easily understand it. We accept comment on interpretation of result and tried to interpret the results in main documents.

-Does not discuss the actual microbiological impact of Salmonella/Shigella on food handlers; the implications for global antimicrobial resistance; nor the limitations of parasite diagnosis by microscopy and study biases. Does not explain why Shigella is 100% sensitive (unusual data requiring serious microbiological interpretation) and does not analyze whether the associated variables have a plausible causal model.

Response: This is very interesting comment we tried to improve in main document

8) The conclusions are partialy coherent, but stating that prevalence is "high" is subjective without a point of comparison or epidemiological criteria. The recommendations are not directly derived from data (e.g., knowledge of hygiene and the impact of training were not measured). Key limitations are not mentioned (only one in Discussion).

Response: - Thank you for your comment. The conclusion and recommendation was updated in main document. To my best knowledge limitation is written in discussion section this why we made it in discussion. However in updated main document we add one limitation not discussed before.

Response for Reviewer 3

1. Introduction section, Re-write it….. use epidemiological term rather than using word dangerous..

Response: - This very interesting suggestion I have changed term to **potential source of infection.**

2. What did you do for the positive cases? Intervention taken has to be explained here.
   Response: - This very interesting question. Food handlers with positive result were linked to Bule hora University teaching hospital for medication and health education was given on personal hygiene. This was done by maintain the privacy of the participants.
3. Would have better include the parasites, bacterias and co-infection with different parasites or with the bacteria including the table. How about the co-infection status?

Response: - Thank you for your suggestion. However, during laboratory analysis we haven't considered it. So, this time we have no data on hand.

4. Italicize all parasites and bacterial names across the document

Response: - Thank you for your comment. We tried to make italic

5. How do the laboratory reagents be a factor for the differences? Did you see the diagnostic techniques employed?
   Response: Oh! Thank you for good view. We have removed this justification.
